# Supplementary material for: DFT Method Used for Prediction of Molecular and Electronic Structures of Mn(VI) Macrocyclic Complexes with Porhyrazine/Phthalocyanine and Two Oxo Ligands
Source: Materials (Basel). 2023 Mar 16;16(6):2394. doi: 10.3390/ma16062394 (PMC10059719; doi:10.3390/ma16062394)
Supplement: Supplementary file 1 [file materials-16-02394-s001.zip › materials-2220583-supplementary.pdf]

NBO Analysis Data for [Mn(P)(O)$_2$]

B3PW91/TZVP

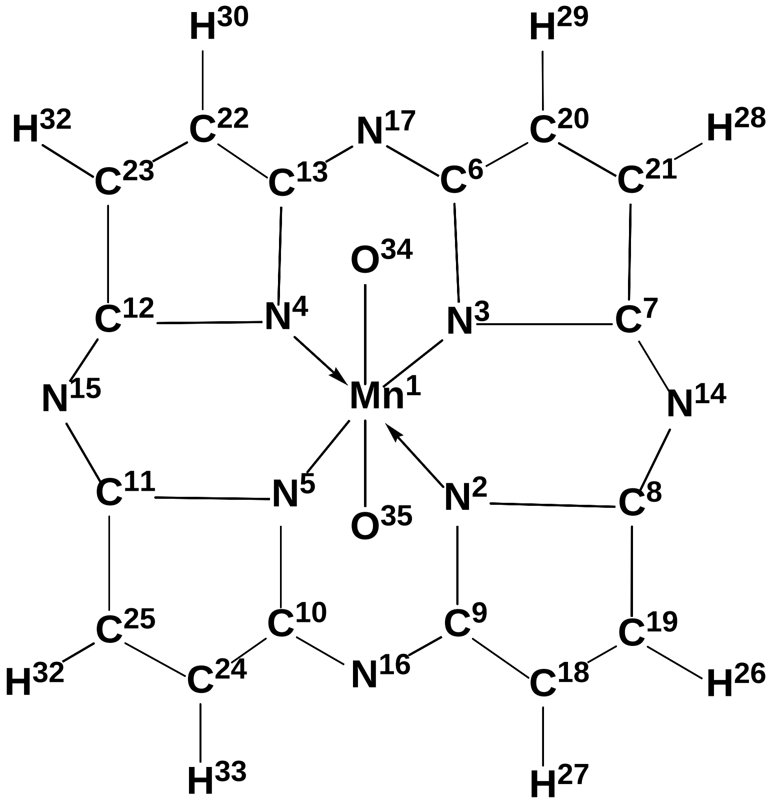

Dipole moment: = 0.1750 Debye

Mulliken charges and spin densities:

1	Mn	0.002241	2.882080
2	N	0.042749	-0.051227
3	N	0.120370	-0.065701
4	N	0.042772	-0.051225
5	N	0.120373	-0.065700
6	C	0.013280	0.009675
7	C	0.013287	0.009675
8	C	0.026398	0.007753
9	C	0.026405	0.007751
10	C	0.013298	0.009673
11	C	0.013270	0.009674
12	C	0.026397	0.007750
13	C	0.026406	0.007750
14	N	-0.003685	-0.000883
15	N	-0.003676	-0.000880
16	N	-0.003684	-0.000881
17	N	-0.003677	-0.000880
18	C	-0.148872	0.016000
19	C	-0.148867	0.015998
20	C	-0.158676	-0.000100
21	C	-0.158664	-0.000100
22	C	-0.148873	0.015998
23	C	-0.148871	0.015997
24	C	-0.158671	-0.000099
25	C	-0.158669	-0.000102
26	H	0.142486	-0.000280
27	H	0.142486	-0.000279
28	H	0.140627	0.000164
29	H	0.140627	0.000164
30	H	0.142486	-0.000280
31	H	0.142485	-0.000279
32	H	0.140626	0.000164
33	H	0.140627	0.000164
34	O	-0.203914	1.107224
35	O	-0.170897	-0.884757

Sum of Mulliken charges = -0.00000 3.00000

ΔE(multipl.=2) = 5.2 kJ/mol
ΔE(multipl.=4) = 0.0 kJ/mol
ΔE(multipl.=6) = 7.8 kJ/mol
Alpha occupied eigenvalues (highest) = -6.5026458 eV
Alpha virtual eigenvalues (lowest) = -3.8298075 eV
Beta occupied eigenvalues (highest) = -6.4748916 eV
Beta virtual eigenvalues (lowest) = -4.4039385 eV

<S**2> = 3.9600

Summary of Natural Population Analysis:

			Natural Population			
Atom	No	Natural Charge	Core	Valence	Rydberg	Total
Mn	1	0.20019	17.99141	6.75253	0.05587	24.79981
N	2	-0.36173	1.99921	5.33603	0.02649	7.36173
N	3	-0.35613	1.99921	5.32938	0.02754	7.35613
N	4	-0.36172	1.99921	5.33602	0.02649	7.36172
N	5	-0.35613	1.99921	5.32938	0.02754	7.35613
C	6	0.42200	1.99926	3.55576	0.02297	5.57800
C	7	0.42199	1.99926	3.55577	0.02297	5.57801
C	8	0.41723	1.99925	3.56066	0.02286	5.58277
C	9	0.41722	1.99925	3.56066	0.02287	5.58278
C	10	0.42199	1.99926	3.55577	0.02297	5.57801
C	11	0.42200	1.99926	3.55576	0.02297	5.57800
C	12	0.41723	1.99925	3.56065	0.02287	5.58277
C	13	0.41723	1.99925	3.56065	0.02287	5.58277
N	14	-0.40666	1.99933	5.39141	0.01592	7.40666
N	15	-0.40666	1.99933	5.39141	0.01592	7.40666
N	16	-0.40666	1.99933	5.39141	0.01592	7.40666
N	17	-0.40666	1.99933	5.39141	0.01592	7.40666
C	18	-0.20446	1.99910	4.19193	0.01343	6.20446
C	19	-0.20446	1.99910	4.19193	0.01343	6.20446

C	20	-0.20570	1.99910	4.19328	0.01333	6.20570
C	21	-0.20570	1.99910	4.19328	0.01333	6.20570
C	22	-0.20446	1.99910	4.19193	0.01343	6.20446
C	23	-0.20446	1.99910	4.19193	0.01343	6.20446
C	24	-0.20570	1.99910	4.19328	0.01333	6.20570
C	25	-0.20570	1.99910	4.19328	0.01333	6.20570
H	26	0.24153	0.00000	0.75712	0.00136	0.75847
H	27	0.24153	0.00000	0.75712	0.00136	0.75847
H	28	0.24128	0.00000	0.75736	0.00136	0.75872
H	29	0.24128	0.00000	0.75736	0.00136	0.75872
H	30	0.24153	0.00000	0.75712	0.00136	0.75847
H	31	0.24153	0.00000	0.75712	0.00136	0.75847
H	32	0.24128	0.00000	0.75736	0.00136	0.75872
H	33	0.24128	0.00000	0.75736	0.00136	0.75872
O	34	-0.36975	1.99995	6.36478	0.00501	8.36975
O	35	-0.41553	1.99995	6.41023	0.00536	8.41553
* Total *		-0.00000	69.97234	130.48841	0.53925	201.00000

NBO Analysis Data for [Mn(P)(O)$_2$]

OPBE/TZVP

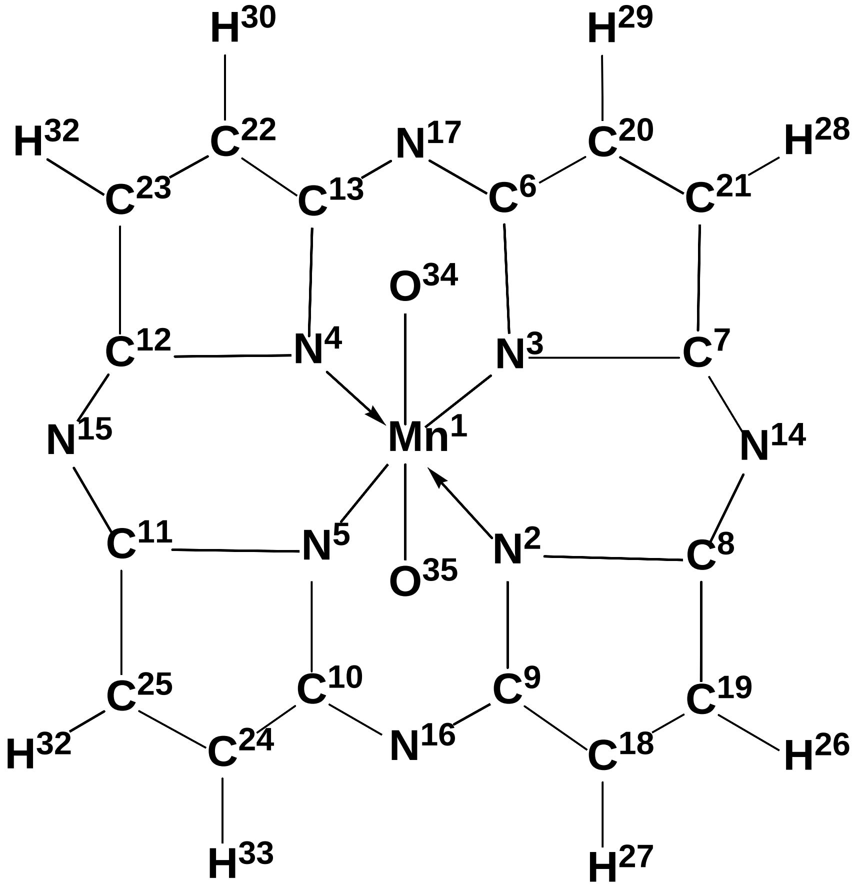

Dipole moment: = 0.2753 Debye

Mulliken charges and spin densities:

1	Mn	-0.599589	1.316333
2	N	0.376846	-0.034543
3	N	0.376839	-0.034536
4	N	0.389331	-0.030591
5	N	0.389287	-0.030583
6	C	-0.135027	0.004033
7	C	-0.111569	0.004387
8	C	-0.111605	0.004387
9	C	-0.135028	0.004032
10	C	-0.106949	0.002744
11	C	-0.132167	0.003085
12	C	-0.132169	0.003084
13	C	-0.106959	0.002744
14	N	0.277085	0.028793
15	N	0.289829	0.035483
16	N	0.282700	0.032365
17	N	0.282714	0.032372
18	C	-0.164012	0.002857
19	C	-0.167530	-0.000064
20	C	-0.164026	0.002860
21	C	-0.167544	-0.000066
22	C	-0.175446	-0.002917
23	C	-0.171254	0.000917
24	C	-0.175447	-0.002918
25	C	-0.171248	0.000921
26	H	0.055161	0.001803
27	H	0.054629	0.001193
28	H	0.054634	0.001192
29	H	0.055164	0.001804
30	H	0.054565	0.000946
31	H	0.054854	0.001709
32	H	0.054860	0.001709
33	H	0.054573	0.000946
34	O	-0.088041	-0.159496
35	O	-0.087460	-0.196981

Sum of Mulliken charges = -0.00000 1.00000

ΔE(multipl.=2) = 0.0 kJ/mol
ΔE(multipl.=4) = 140.7 kJ/mol
ΔE(multipl.=6) = 118.3 kJ/mol
Alpha occupied eigenvalues (highest) = -5.95899 eV
Alpha virtual eigenvalues (lowest) = -4.6180812 eV
Beta occupied eigenvalues (highest) = -5.9573574 eV
Beta virtual eigenvalues (lowest) = -5.512746 eV

<S**2>= 0.8218

Summary of Natural Population Analysis:

Atom	No	Natural Charge	Natural Population Core	Valence	Rydberg	Total
Mn	1	-0.07148	17.99045	7.04757	0.03345	25.07148
N	2	-0.30513	1.99920	5.27920	0.02672	7.30513
N	3	-0.30512	1.99920	5.27920	0.02672	7.30512
N	4	-0.30237	1.99919	5.27524	0.02794	7.30237
N	5	-0.30238	1.99919	5.27526	0.02794	7.30238
C	6	0.33292	1.99927	3.64768	0.02013	5.66708
C	7	0.33315	1.99927	3.64728	0.02031	5.66685
C	8	0.33315	1.99927	3.64728	0.02031	5.66685
C	9	0.33292	1.99927	3.64768	0.02013	5.66708
C	10	0.33403	1.99926	3.64616	0.02055	5.66597
C	11	0.33534	1.99926	3.64512	0.02028	5.66466
C	12	0.33534	1.99926	3.64512	0.02028	5.66466
C	13	0.33403	1.99926	3.64616	0.02055	5.66597
N	14	-0.34755	1.99934	5.33387	0.01434	7.34755
N	15	-0.34424	1.99933	5.33081	0.01411	7.34424
N	16	-0.34515	1.99933	5.33159	0.01422	7.34515
N	17	-0.34514	1.99933	5.33159	0.01422	7.34514
C	18	-0.21739	1.99911	4.20667	0.01161	6.21739
C	19	-0.22101	1.99911	4.21030	0.01160	6.22101
C	20	-0.21739	1.99911	4.20667	0.01161	6.21739
C	21	-0.22101	1.99911	4.21030	0.01160	6.22101

C	22	-0.22268	1.99911	4.21206	0.01151	6.22268
C	23	-0.21848	1.99911	4.20784	0.01153	6.21848
C	24	-0.22268	1.99911	4.21207	0.01151	6.22268
C	25	-0.21847	1.99911	4.20783	0.01152	6.21847
H	26	0.24492	0.00000	0.75353	0.00154	0.75508
H	27	0.24482	0.00000	0.75363	0.00154	0.75518
H	28	0.24482	0.00000	0.75363	0.00154	0.75518
H	29	0.24492	0.00000	0.75353	0.00154	0.75508
H	30	0.24414	0.00000	0.75430	0.00156	0.75586
H	31	0.24428	0.00000	0.75417	0.00155	0.75572
H	32	0.24428	0.00000	0.75417	0.00155	0.75572
H	33	0.24414	0.00000	0.75430	0.00156	0.75586
O	34	-0.09992	1.99994	6.09240	0.00759	8.09992
O	35	-0.09961	1.99994	6.09211	0.00756	8.09961
* Total *		0.00000	69.97144	130.54632	0.48224	201.00000

NATURAL POPULATIONS: Natural atomic orbital occupancies

NAO	Atom	No	lang	Type(AO)	Occupancy
1	Mn	1	S	Cor(1S)	2.00000
2	Mn	1	S	Cor(2S)	2.00000
3	Mn	1	S	Cor(3S)	1.99472
4	Mn	1	S	Val(4S)	0.30635
5	Mn	1	S	Ryd(5S)	0.00202
6	Mn	1	S	Ryd(6S)	0.00038
7	Mn	1	px	Cor(2p)	2.00000
8	Mn	1	px	Cor(3p)	1.99833
9	Mn	1	px	Val(4p)	0.26705
10	Mn	1	px	Ryd(5p)	0.00084
11	Mn	1	py	Cor(2p)	2.00000
12	Mn	1	py	Cor(3p)	1.99824
13	Mn	1	py	Val(4p)	0.26774
14	Mn	1	py	Ryd(5p)	0.00083
15	Mn	1	pz	Cor(2p)	2.00000
16	Mn	1	pz	Cor(3p)	1.99917

17	Mn	1	pz	Val(4p)	0.25974
18	Mn	1	pz	Ryd(5p)	0.00207
19	Mn	1	dxy	Val(3d)	1.14074
20	Mn	1	dxy	Ryd(4d)	0.00514
21	Mn	1	dxy	Ryd(5d)	0.00004
22	Mn	1	dxz	Val(3d)	1.18693
23	Mn	1	dxz	Ryd(4d)	0.00102
24	Mn	1	dxz	Ryd(5d)	0.00007
25	Mn	1	dyz	Val(3d)	1.18505
26	Mn	1	dyz	Ryd(4d)	0.00166
27	Mn	1	dyz	Ryd(5d)	0.00007
28	Mn	1	dx2y2	Val(3d)	1.23347
29	Mn	1	dx2y2	Ryd(4d)	0.00784
30	Mn	1	dx2y2	Ryd(5d)	0.00037
31	Mn	1	dz2	Val(3d)	1.20049
32	Mn	1	dz2	Ryd(4d)	0.01104
33	Mn	1	dz2	Ryd(5d)	0.00006

NBO Analysis Data for [Mn(Pc)(O)$_2$]

B3PW91/TZVP

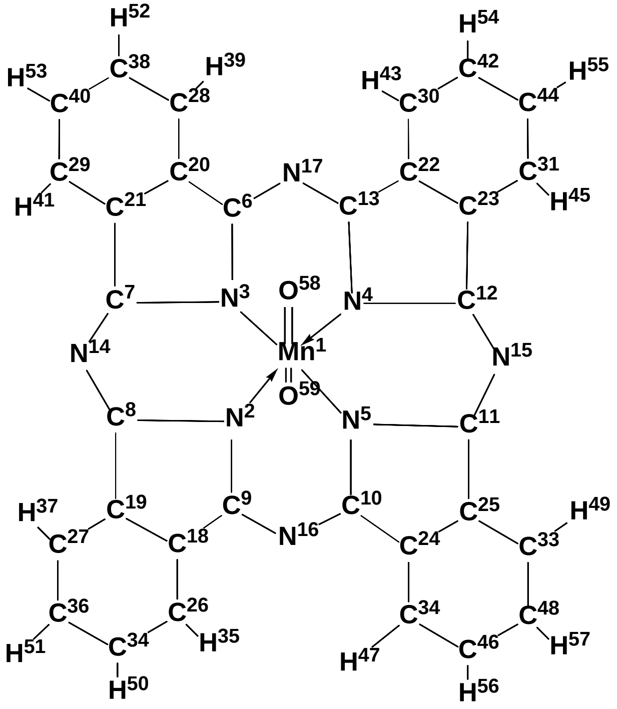

Dipole moment: = 0.0012 Debye

Mulliken charges and spin densities:

1	Mn	-0.103203	0.017054
2	N	0.159543	-0.069772
3	N	0.159543	-0.069772
4	N	0.159543	-0.069772
5	N	0.159543	-0.069772
6	C	0.015947	0.171054
7	C	0.015927	0.171053
8	C	0.015947	0.171054
9	C	0.015927	0.171053
10	C	0.015947	0.171054
11	C	0.015927	0.171053
12	C	0.015947	0.171054
13	C	0.015927	0.171053
14	N	-0.039379	-0.073141
15	N	-0.039379	-0.073141
16	N	-0.039379	-0.073141
17	N	-0.039379	-0.073141
18	C	-0.010303	-0.026789
19	C	-0.010314	-0.026788
20	C	-0.010314	-0.026788
21	C	-0.010303	-0.026789
22	C	-0.010303	-0.026789
23	C	-0.010314	-0.026788
24	C	-0.010314	-0.026788
25	C	-0.010303	-0.026789
26	C	-0.148546	0.041124
27	C	-0.148552	0.041123
28	C	-0.148552	0.041124
29	C	-0.148546	0.041124
30	C	-0.148546	0.041124
31	C	-0.148552	0.041123
32	C	-0.148552	0.041124
33	C	-0.148546	0.041124
34	C	-0.073421	0.013769
35	H	0.124899	-0.001354
36	C	-0.073420	0.013770
37	H	0.124898	-0.001354
38	C	-0.073420	0.013770
39	H	0.124898	-0.001354
40	C	-0.073421	0.013769
41	H	0.124899	-0.001354
42	C	-0.073421	0.013769
43	H	0.124899	-0.001354
44	C	-0.073420	0.013770
45	H	0.124898	-0.001354
46	C	-0.073420	0.013770
47	H	0.124898	-0.001354
48	C	-0.073421	0.013769
49	H	0.124899	-0.001354
50	H	0.116558	-0.001193
51	H	0.116558	-0.001193
52	H	0.116558	-0.001193
53	H	0.116558	-0.001193
54	H	0.116558	-0.001193
55	H	0.116558	-0.001193
56	H	0.116558	-0.001193
57	H	0.116558	-0.001193
58	O	-0.289174	-0.009200
59	O	-0.289202	-0.009102

Sum of Mulliken charges = -0.00000 1.00000

ΔE(multipl.=2) = 0.0 kJ/mol
ΔE(multipl.=4) = 47.4 kJ/mol
ΔE(multipl.=6) = 147.9 kJ/mol
Alpha occupied eigenvalues (highest) = -5.7551871 eV
Alpha virtual eigenvalues (lowest) = -3.2279223 eV
Beta occupied eigenvalues (highest) = -6.2890473 eV
Beta virtual eigenvalues (lowest) = -4.5644775 eV

<S**2>= 0.7803

Summary of Natural Population Analysis:

Atom	No	Natural Charge	Natural Population Core	Valence	Rydberg	Total
Mn	1	-0.15600	17.99051	7.13208	0.03341	25.15600
N	2	-0.31801	1.99922	5.29114	0.02766	7.31801
N	3	-0.31801	1.99922	5.29113	0.02766	7.31801
N	4	-0.31801	1.99922	5.29114	0.02766	7.31801
N	5	-0.31801	1.99922	5.29114	0.02766	7.31801
C	6	0.45083	1.99921	3.52743	0.02252	5.54917
C	7	0.45083	1.99921	3.52743	0.02252	5.54917
C	8	0.45083	1.99921	3.52743	0.02252	5.54917
C	9	0.45083	1.99921	3.52743	0.02252	5.54917
C	10	0.45083	1.99921	3.52743	0.02252	5.54917
C	11	0.45083	1.99921	3.52743	0.02252	5.54917
C	12	0.45083	1.99921	3.52743	0.02252	5.54917
C	13	0.45083	1.99921	3.52743	0.02252	5.54917
N	14	-0.44423	1.99933	5.42906	0.01584	7.44423
N	15	-0.44423	1.99933	5.42906	0.01584	7.44423
N	16	-0.44423	1.99933	5.42906	0.01584	7.44423
N	17	-0.44423	1.99933	5.42906	0.01584	7.44423
C	18	-0.08245	1.99902	4.06624	0.01719	6.08245
C	19	-0.08245	1.99902	4.06624	0.01719	6.08245
C	20	-0.08245	1.99902	4.06624	0.01719	6.08245

C	21	-0.08245	1.99902	4.06624	0.01719	6.08245
C	22	-0.08245	1.99902	4.06624	0.01719	6.08245
C	23	-0.08245	1.99902	4.06624	0.01719	6.08245
C	24	-0.08245	1.99902	4.06624	0.01719	6.08245
C	25	-0.08245	1.99902	4.06624	0.01719	6.08245
C	26	-0.15897	1.99912	4.14620	0.01365	6.15897
C	27	-0.15897	1.99912	4.14620	0.01365	6.15897
C	28	-0.15897	1.99912	4.14620	0.01365	6.15897
C	29	-0.15897	1.99912	4.14620	0.01365	6.15897
C	30	-0.15897	1.99912	4.14620	0.01365	6.15897
C	31	-0.15897	1.99912	4.14620	0.01365	6.15897
C	32	-0.15897	1.99912	4.14620	0.01365	6.15897
C	33	-0.15897	1.99912	4.14620	0.01365	6.15897
C	34	-0.19789	1.99925	4.18474	0.01390	6.19789
H	35	0.23245	0.00000	0.76588	0.00167	0.76755
C	36	-0.19789	1.99925	4.18474	0.01390	6.19789
H	37	0.23245	0.00000	0.76588	0.00167	0.76755
C	38	-0.19789	1.99925	4.18474	0.01390	6.19789
H	39	0.23245	0.00000	0.76588	0.00167	0.76755
C	40	-0.19789	1.99925	4.18474	0.01390	6.19789
H	41	0.23245	0.00000	0.76588	0.00167	0.76755
C	42	-0.19789	1.99925	4.18474	0.01390	6.19789
H	43	0.23245	0.00000	0.76588	0.00167	0.76755
C	44	-0.19789	1.99925	4.18474	0.01390	6.19789
H	45	0.23245	0.00000	0.76588	0.00167	0.76755
C	46	-0.19789	1.99925	4.18474	0.01390	6.19789
H	47	0.23245	0.00000	0.76588	0.00167	0.76755
C	48	-0.19789	1.99925	4.18474	0.01390	6.19789
H	49	0.23245	0.00000	0.76588	0.00167	0.76755
H	50	0.21872	0.00000	0.78016	0.00112	0.78128
H	51	0.21872	0.00000	0.78016	0.00112	0.78128
H	52	0.21872	0.00000	0.78016	0.00112	0.78128
H	53	0.21872	0.00000	0.78016	0.00112	0.78128
H	54	0.21872	0.00000	0.78016	0.00112	0.78128
H	55	0.21872	0.00000	0.78016	0.00112	0.78128
H	56	0.21872	0.00000	0.78016	0.00112	0.78128
H	57	0.21872	0.00000	0.78016	0.00112	0.78128

O	58	-0.24829	1.99993	6.24179	0.00656	8.24829
O	59	-0.24835	1.99993	6.24185	0.00656	8.24835
* Total *		0.00000	101.95737	202.26172	0.78092	305.00000

NATURAL POPULATIONS: Natural atomic orbital occupancies

NAO	Atom	No	lang	Type(AO)	Occupancy
1	Mn	1	S	Cor(1S)	2.00000
2	Mn	1	S	Cor(2S)	2.00000
3	Mn	1	S	Cor(3S)	1.99393
4	Mn	1	S	Val(4S)	0.28595
5	Mn	1	S	Ryd(5S)	0.00093
6	Mn	1	S	Ryd(6S)	0.00038
7	Mn	1	px	Cor(2p)	2.00000
8	Mn	1	px	Cor(3p)	1.99840
9	Mn	1	px	Val(4p)	0.26191
10	Mn	1	px	Ryd(5p)	0.00045
11	Mn	1	py	Cor(2p)	2.00000
12	Mn	1	py	Cor(3p)	1.99840
13	Mn	1	py	Val(4p)	0.26191
14	Mn	1	py	Ryd(5p)	0.00045
15	Mn	1	pz	Cor(2p)	2.00000
16	Mn	1	pz	Cor(3p)	1.99980
17	Mn	1	pz	Val(4p)	0.25564
18	Mn	1	pz	Ryd(5p)	0.00240
19	Mn	1	dxy	Val(3d)	1.97925
20	Mn	1	dxy	Ryd(4d)	0.01073
21	Mn	1	dxy	Ryd(5d)	0.00045
22	Mn	1	dxz	Val(3d)	1.04467
23	Mn	1	dxz	Ryd(4d)	0.00012
24	Mn	1	dxz	Ryd(5d)	0.00007
25	Mn	1	dyz	Val(3d)	1.04467
26	Mn	1	dyz	Ryd(4d)	0.00012
27	Mn	1	dyz	Ryd(5d)	0.00007
28	Mn	1	dx2y2	Val(3d)	0.86537

29	Mn	1	dx2y2	Ryd(4d)	0.00516
30	Mn	1	dx2y2	Ryd(5d)	0.00005
31	Mn	1	dz2	Val(3d)	1.13271
32	Mn	1	dz2	Ryd(4d)	0.01200
33	Mn	1	dz2	Ryd(5d)	0.00004

NBO Analysis Data for [Mn(Pc)(O)$_2$]

OPBE/TZVP

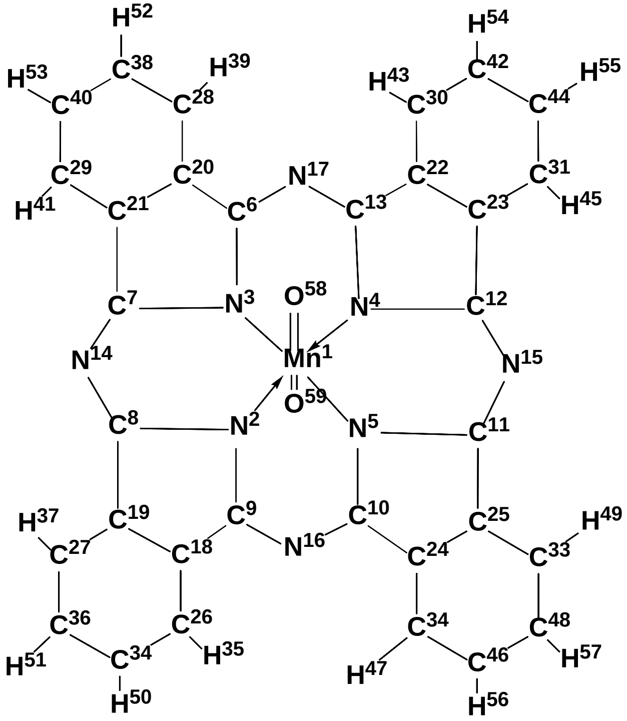

Dipole moment: = 0.0346 Debye

Mulliken charges and spin densities:

1	Mn	-0.515772	0.641513
2	N	0.406494	-0.038489
3	N	0.404026	-0.038434
4	N	0.406419	-0.038482
5	N	0.404047	-0.038427
6	C	-0.160560	0.071887
7	C	-0.160405	0.071879
8	C	-0.163653	0.071959
9	C	-0.163146	0.071942
10	C	-0.160319	0.071850
11	C	-0.160466	0.071857
12	C	-0.163152	0.071943
13	C	-0.163649	0.071959
14	N	0.257526	-0.002215
15	N	0.257512	-0.002204
16	N	0.257499	-0.002199
17	N	0.257539	-0.002220
18	C	0.177087	-0.011284
19	C	0.177443	-0.011290
20	C	0.176623	-0.011317
21	C	0.176493	-0.011314
22	C	0.177412	-0.011293
23	C	0.177064	-0.011286
24	C	0.176413	-0.011309
25	C	0.176538	-0.011312
26	C	-0.348733	0.022312
27	C	-0.348620	0.022321
28	C	-0.349270	0.022312
29	C	-0.349258	0.022311
30	C	-0.348621	0.022320
31	C	-0.348731	0.022311
32	C	-0.349264	0.022302
33	C	-0.349279	0.022303
34	C	-0.013417	0.007353
35	H	0.058763	-0.000811
36	C	-0.013458	0.007340
37	H	0.058783	-0.000812
38	C	-0.013336	0.007336
39	H	0.058696	-0.000812
40	C	-0.013353	0.007337
41	H	0.058692	-0.000812
42	C	-0.013461	0.007339
43	H	0.058779	-0.000812
44	C	-0.013420	0.007352
45	H	0.058759	-0.000811
46	C	-0.013355	0.007333
47	H	0.058687	-0.000811
48	C	-0.013337	0.007333
49	H	0.058691	-0.000811
50	H	0.058830	-0.000725
51	H	0.058833	-0.000725
52	H	0.058809	-0.000724
53	H	0.058807	-0.000724
54	H	0.058830	-0.000725
55	H	0.058827	-0.000725
56	H	0.058806	-0.000724
57	H	0.058808	-0.000724
58	O	-0.149094	-0.089465
59	O	-0.147407	-0.099176

Sum of Mulliken charges = -0.00000 1.00000

ΔE(multipl.=2) = 0.0 kJ/mol
ΔE(multipl.=4) = 96.5 kJ/mol
ΔE(multipl.=6) = 126.1 kJ/mol
Alpha occupied eigenvalues (highest) = -5.1051402 eV
Alpha virtual eigenvalues (lowest) = -3.6039645 eV
Beta occupied eigenvalues (highest) = -5.1364317 eV
Beta virtual eigenvalues (lowest) = -4.8594339 eV

<S**2>= 0.7668

Summary of Natural Population Analysis:

		Natural	Natural Population			
Atom	No	Charge	Core	Valence	Rydberg	Total
Mn	1	-0.17524	17.99060	7.15584	0.02879	25.17524
N	2	-0.28160	1.99923	5.25562	0.02675	7.28160
N	3	-0.28172	1.99923	5.25573	0.02676	7.28172
N	4	-0.28165	1.99923	5.25567	0.02675	7.28165
N	5	-0.28170	1.99923	5.25572	0.02676	7.28170
C	6	0.39041	1.99921	3.59035	0.02003	5.60959
C	7	0.39041	1.99921	3.59035	0.02003	5.60959
C	8	0.39046	1.99921	3.59029	0.02004	5.60954
C	9	0.39046	1.99921	3.59030	0.02004	5.60954
C	10	0.39040	1.99921	3.59036	0.02003	5.60960
C	11	0.39041	1.99921	3.59035	0.02003	5.60959
C	12	0.39046	1.99921	3.59030	0.02004	5.60954
C	13	0.39047	1.99921	3.59029	0.02004	5.60953
N	14	-0.38931	1.99933	5.37554	0.01444	7.38931
N	15	-0.38930	1.99933	5.37554	0.01444	7.38930
N	16	-0.38930	1.99933	5.37553	0.01444	7.38930
N	17	-0.38931	1.99933	5.37555	0.01444	7.38931
C	18	-0.08096	1.99905	4.06651	0.01540	6.08096
C	19	-0.08097	1.99905	4.06652	0.01540	6.08097
C	20	-0.08096	1.99905	4.06651	0.01541	6.08096

C	21	-0.08097	1.99905	4.06652	0.01541	6.08097
C	22	-0.08097	1.99905	4.06652	0.01540	6.08097
C	23	-0.08096	1.99905	4.06651	0.01540	6.08096
C	24	-0.08097	1.99905	4.06651	0.01541	6.08097
C	25	-0.08096	1.99905	4.06650	0.01541	6.08096
C	26	-0.16900	1.99913	4.15805	0.01182	6.16900
C	27	-0.16900	1.99913	4.15805	0.01182	6.16900
C	28	-0.16901	1.99913	4.15807	0.01182	6.16901
C	29	-0.16902	1.99913	4.15807	0.01182	6.16902
C	30	-0.16901	1.99913	4.15806	0.01182	6.16901
C	31	-0.16901	1.99913	4.15806	0.01182	6.16901
C	32	-0.16902	1.99913	4.15807	0.01182	6.16902
C	33	-0.16902	1.99913	4.15807	0.01182	6.16902
C	34	-0.20633	1.99926	4.19527	0.01180	6.20633
H	35	0.23664	0.00000	0.76151	0.00185	0.76336
C	36	-0.20633	1.99926	4.19528	0.01180	6.20633
H	37	0.23664	0.00000	0.76151	0.00185	0.76336
C	38	-0.20633	1.99926	4.19527	0.01180	6.20633
H	39	0.23663	0.00000	0.76151	0.00185	0.76337
C	40	-0.20633	1.99926	4.19527	0.01180	6.20633
H	41	0.23663	0.00000	0.76152	0.00185	0.76337
C	42	-0.20634	1.99926	4.19528	0.01180	6.20634
H	43	0.23664	0.00000	0.76151	0.00185	0.76336
C	44	-0.20633	1.99926	4.19527	0.01180	6.20633
H	45	0.23664	0.00000	0.76151	0.00185	0.76336
C	46	-0.20633	1.99926	4.19527	0.01180	6.20633
H	47	0.23663	0.00000	0.76152	0.00185	0.76337
C	48	-0.20633	1.99926	4.19527	0.01180	6.20633
H	49	0.23663	0.00000	0.76151	0.00185	0.76337
H	50	0.22405	0.00000	0.77471	0.00124	0.77595
H	51	0.22405	0.00000	0.77471	0.00124	0.77595
H	52	0.22405	0.00000	0.77471	0.00124	0.77595
H	53	0.22405	0.00000	0.77471	0.00124	0.77595
H	54	0.22405	0.00000	0.77471	0.00124	0.77595
H	55	0.22405	0.00000	0.77471	0.00124	0.77595
H	56	0.22405	0.00000	0.77471	0.00124	0.77595
H	57	0.22405	0.00000	0.77471	0.00124	0.77595

O	58	-0.14935	1.99994	6.14228	0.00714	8.14935
O	59	-0.15003	1.99994	6.14299	0.00711	8.15003
* Total *		0.00000	101.95783	202.33711	0.70506	305.00000

NATURAL POPULATIONS: Natural atomic orbital occupancies

NAO	Atom	No	lang	Type(AO)	Occupancy
1	Mn	1	S	Cor(1S)	2.00000
2	Mn	1	S	Cor(2S)	2.00000
3	Mn	1	S	Cor(3S)	1.99423
4	Mn	1	S	Val(4S)	0.29736
5	Mn	1	S	Ryd(5S)	0.00151
6	Mn	1	S	Ryd(6S)	0.00039
7	Mn	1	px	Cor(2p)	2.00000
8	Mn	1	px	Cor(3p)	1.99829
9	Mn	1	px	Val(4p)	0.26025
10	Mn	1	px	Ryd(5p)	0.00059
11	Mn	1	py	Cor(2p)	2.00000
12	Mn	1	py	Cor(3p)	1.99829
13	Mn	1	py	Val(4p)	0.26027
14	Mn	1	py	Ryd(5p)	0.00059
15	Mn	1	pz	Cor(2p)	2.00000
16	Mn	1	pz	Cor(3p)	1.99980
17	Mn	1	pz	Val(4p)	0.25387
18	Mn	1	pz	Ryd(5p)	0.00198
19	Mn	1	dxy	Val(3d)	1.60793
20	Mn	1	dxy	Ryd(4d)	0.00707
21	Mn	1	dxy	Ryd(5d)	0.00038
22	Mn	1	dxz	Val(3d)	1.13347
23	Mn	1	dxz	Ryd(4d)	0.00049
24	Mn	1	dxz	Ryd(5d)	0.00006
25	Mn	1	dyz	Val(3d)	1.13356
26	Mn	1	dyz	Ryd(4d)	0.00048
27	Mn	1	dyz	Ryd(5d)	0.00006
28	Mn	1	dx2y2	Val(3d)	1.03024

29	Mn	1	dx2y2	Ryd(4d)	0.00421
30	Mn	1	dx2y2	Ryd(5d)	0.00006
31	Mn	1	dz2	Val(3d)	1.17889
32	Mn	1	dz2	Ryd(4d)	0.01087
33	Mn	1	dz2	Ryd(5d)	0.00006

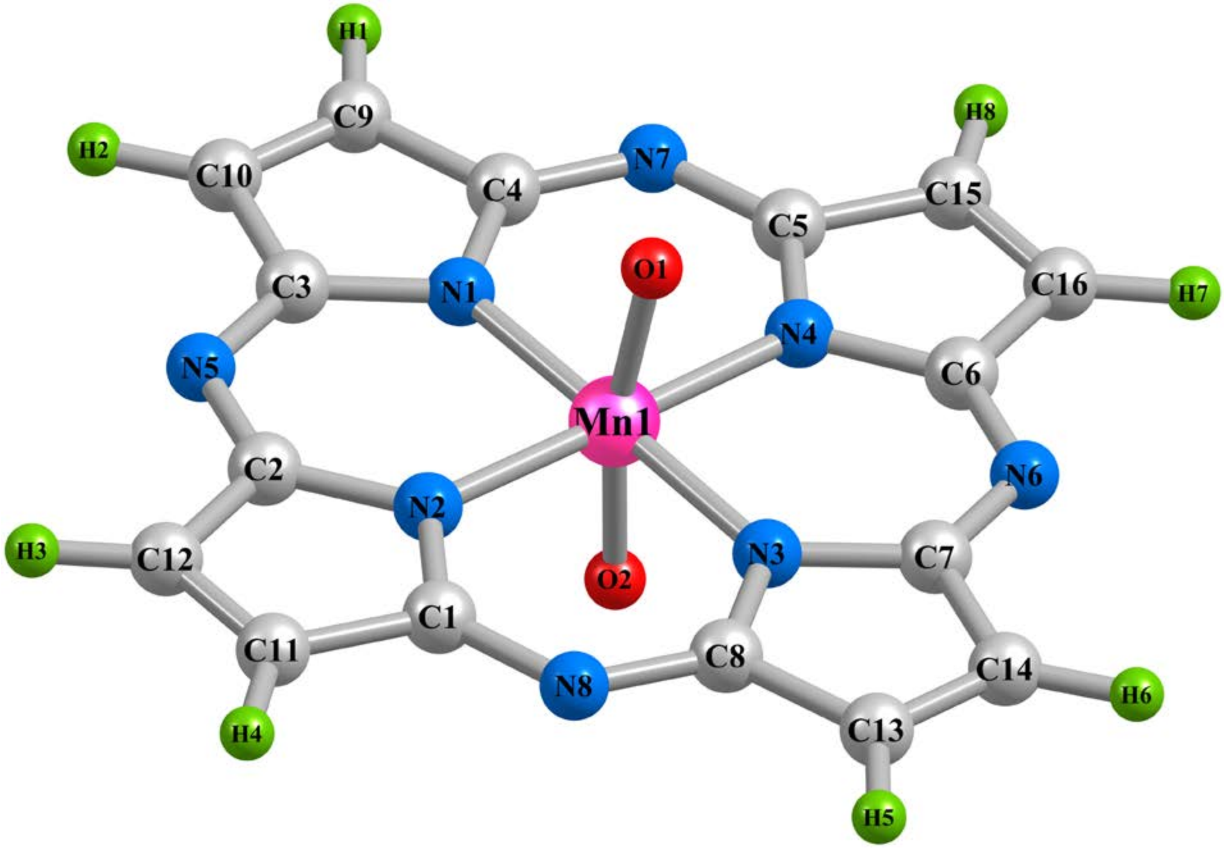

(a)

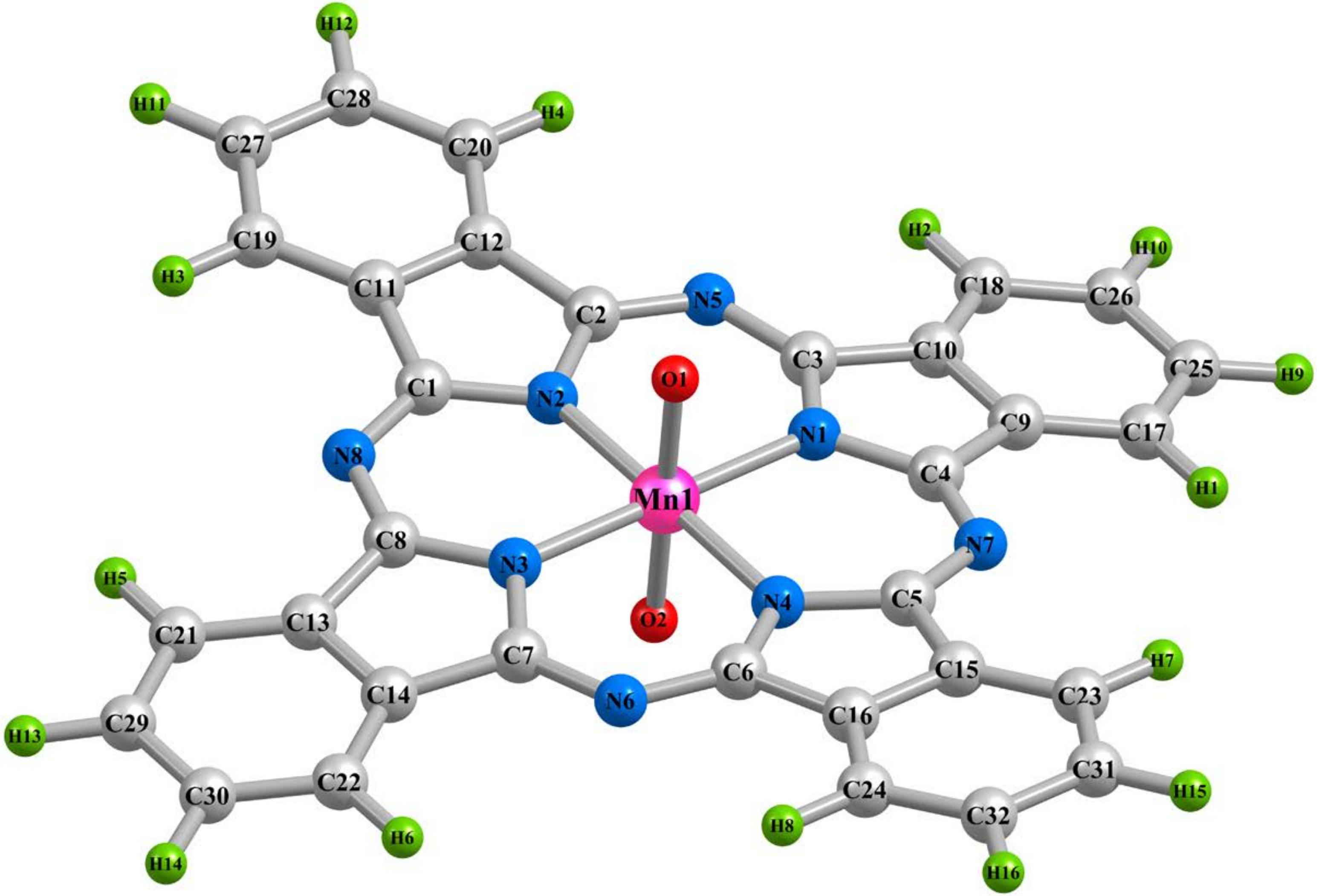

(b)

Figure S1. Molecular structures of the [Mn(**P**)(O)$_2$] (a) and [Mn(**Pc**)(O)$_2$] (b) complexes obtained as a result of DFT OPBE/TZVP quantum-chemical calculation.
